# Metabolic analyses elucidate non-trivial gene targets for amplifying dihydroartemisinic acid production in yeast

**DOI:** 10.3389/fmicb.2013.00200

**Published:** 2013-07-26

**Authors:** Ashish Misra, Matthew F. Conway, Joseph Johnnie, Tabish M. Qureshi, Bao Lige, Anne M. Derrick, Eddy C. Agbo, Ganesh Sriram

**Affiliations:** ^1^Department of Chemical and Biomolecular Engineering, University of MarylandCollege Park, MD, USA; ^2^Institute for Systems Engineering, University of MarylandCollege Park, MD, USA; ^3^Fyodor BiotechnologiesBaltimore, MD, USA

**Keywords:** artemisinin, metabolic engineering, metabolic pathway, extreme pathway, isotope labeling, metabolic flux analysis, flux balance analysis, minimization of metabolic adjustment

## Abstract

Synthetic biology enables metabolic engineering of industrial microbes to synthesize value-added molecules. In this, a major challenge is the efficient redirection of carbon to the desired metabolic pathways. Pinpointing strategies toward this goal requires an in-depth investigation of the metabolic landscape of the organism, particularly primary metabolism, to identify precursor and cofactor availability for the target compound. The potent antimalarial therapeutic artemisinin and its precursors are promising candidate molecules for production in microbial hosts. Recent advances have demonstrated the production of artemisinin precursors in engineered yeast strains as an alternative to extraction from plants. We report the application of *in silico* and *in vivo* metabolic pathway analyses to identify metabolic engineering targets to improve the yield of the direct artemisinin precursor dihydroartemisinic acid (DHA) in yeast. First, *in silico* extreme pathway (ExPa) analysis identified NADPH-malic enzyme and the oxidative pentose phosphate pathway (PPP) as mechanisms to meet NADPH demand for DHA synthesis. Next, we compared key DHA-synthesizing ExPas to the metabolic flux distributions obtained from *in vivo* ^13^C metabolic flux analysis of a DHA-synthesizing strain. This comparison revealed that knocking out ethanol synthesis and overexpressing glucose-6-phosphate dehydrogenase in the oxidative PPP (gene *YNL241C*) or the NADPH-malic enzyme ME2 (*YKL029C*) are vital steps toward overproducing DHA. Finally, we employed *in silico* flux balance analysis and minimization of metabolic adjustment on a yeast genome-scale model to identify gene knockouts for improving DHA yields. The best strategy involved knockout of an oxaloacetate transporter (*YKL120W*) and an aspartate aminotransferase (*YKL106W*), and was predicted to improve DHA yields by 70-fold. Collectively, our work elucidates multiple non-trivial metabolic engineering strategies for improving DHA yield in yeast.

## INTRODUCTION

**FIGURE 1:**
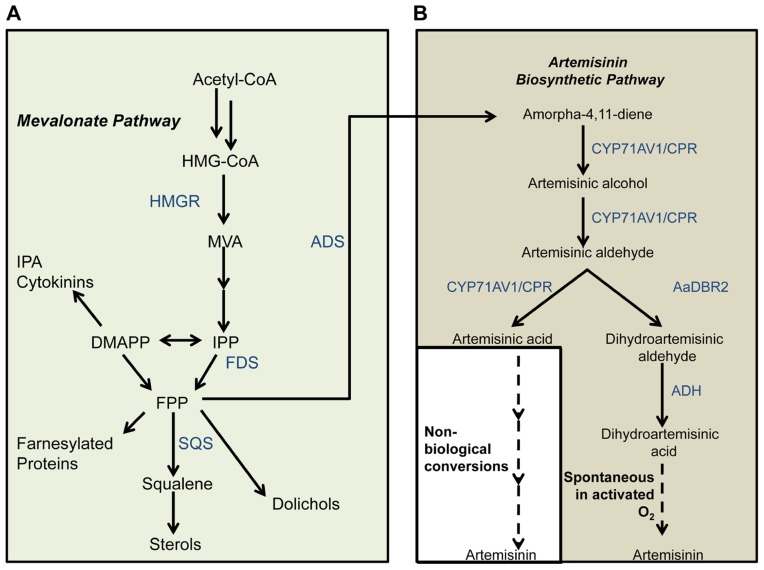
**Artemisinin precursor synthesis pathways in yeast.** In the native isoprenoid biosynthesis pathway in yeast **(A)**, IPP synthesized via the MVA pathway is converted to FPP. HMGR is a key enzyme in the isoprenoid biosynthetic pathway that feeds the artemisinin precursor synthesis pathway **(B)**. Steps that are not known to be catalyzed by enzymes are depicted with dashed lines. Enzyme and metabolite name abbreviations: AaDBR2, *A. annua* double bond reductase; ADH, (dihydroartemisinic) aldehyde dehydrogenase; ADS, amorpha-4,11-diene synthase; CYP71AV1, cytochrome P450 monooxygenase; CPR, *A. annua* cytochrome P450 reductase; CoA, coenzyme A; DMAPP, dimethylallyl diphosphate; FDS, farnesyl diphosphate synthase; FPP, farnesyl pyrophosphate; HMG-CoA, 3-hydroxy-3-methylglutaryl-CoA; HMGR, HMG-CoA reductase; IPA, isopentenyladenine; IPP, isopentenyl diphosphate; MVA, mevalonate; SQS, squalene synthase. Adapted from Zhang et al. (2008) and Teoh et al. (2006).

Artemisinin-based combination therapy (ACT) is currently the most commonly used treatment for malaria (Weathers et al., 2006; Eastman and Fidock, 2009; Price and Douglas, 2009), an infectious disease that is widespread particularly in regions of Africa, Asia, and South America (Feachem et al., 2010; O’Meara et al., 2010). Artemisinin, the primary component of ACT, is a naturally produced sesquiterpene lactone endoperoxide. Artemisinin and its derivatives have also been found to exhibit anti-cancer properties (Ferreira et al., 2010) such as inducing apoptosis in lung cancer cells (Gao et al., 2013) and preventing cell proliferation in breast cancer cells (Ba et al., 2012; Zhang et al., 2013). In nature, artemisinin is synthesized by the plant *Artemisia annua* through conversion of the intermediate sesquiterpene farnesyl pyrophosphate (FPP; Bertea et al., 2005; Liu et al., 2011). The precursor FPP is synthesized from the primary metabolite acetyl-coenzyme A (CoA) via the mevalonate (MVA) pathway, or from glyceraldehyde-3-phosphate (GAP) and pyruvate via the methylerythritol phosphate (MEP) pathway (**Figure 1**). *A. annua* has been the primary source for meeting almost all the worldwide demand for artemisinin, despite producing artemisinin up to less than 1.0–1.5% of its dry weight (Kindermans et al., 2007; Covello, 2008). Not surprisingly, the price of artemisinin has varied substantially from a lower bound of US$150–170 kg^-1^ to an upper bound of US$1,100–1,500 kg^-1^ (World Health Organization, 2010), partially due to variability in the cultivation of *A. annua*. Therefore, it is necessary to explore avenues for reliable production of artemisinin that offer this drug at the minimal possible cost to developing countries (Covello, 2008). To achieve this goal, researchers have demonstrated the synthesis of artemisinin precursors in microbes (Ro et al., 2006; Zhang et al., 2008; Westfall et al., 2012; Paddon et al., 2013) or plants (e.g., Zhang et al., 2011) engineered to express genes from the *A. annua* artemisinin pathway, thereby enabling conversion of endogenously produced FPP to artemisinin precursors. Orthogonally, chemical syntheses for artemisinin from starting materials ranging from natural terpenoids (reviewed in Covello, 2008) to cyclohexenone (e.g., Zhu and Cook, 2012) have been reported.

The first applications of synthetic biology to artemisinin production were those of Keasling and colleagues (Ro et al., 2006) and of Covello and colleagues (Teoh et al., 2006). Both groups reported the engineering of *Saccharomyces cerevisiae* to produce artemisinic acid (AA), which can be converted to artemisinin through a sequence of chemical steps. The breakthrough work by Keasling and colleagues was followed by several reports of significantly improved AA titers from the same group (Westfall et al., 2012; Paddon et al., 2013; see detailed description below). Independently, Covello and colleagues demonstrated the production of dihydroartemisinic acid (DHA) in *S. cerevisiae* (Zhang et al., 2008). As an artemisinin precursor, DHA is preferable to AA for multiple reasons. Firstly, DHA can be oxidized to artemisinin spontaneously without the involvement of enzymes (Sy and Brown, 2002; Brown and Sy, 2004). *In planta* artemisinin biosynthesis is hypothesized to proceed through DHA via this mechanism (Bertea et al., 2005), circumstantial evidence for which comes from the observation that DHA-rich chemotypes of *A. annua* exhibit significantly higher artemisinin production than AA-rich chemotypes (Wallaart et al., 2000; Rydén et al., 2010). Secondly, semi-synthetic routes to artemisinin production have relied upon DHA as the starting material (e.g., Lévesque and Seeberger, 2012; Westfall et al., 2012). Thus, the possibility of engineering DHA biosynthesis in microbes opens up an alternative route for artemisinin synthesis in yeast, which has the potential to be executed completely *in vivo* (Zhang et al., 2008).

**FIGURE 2:**
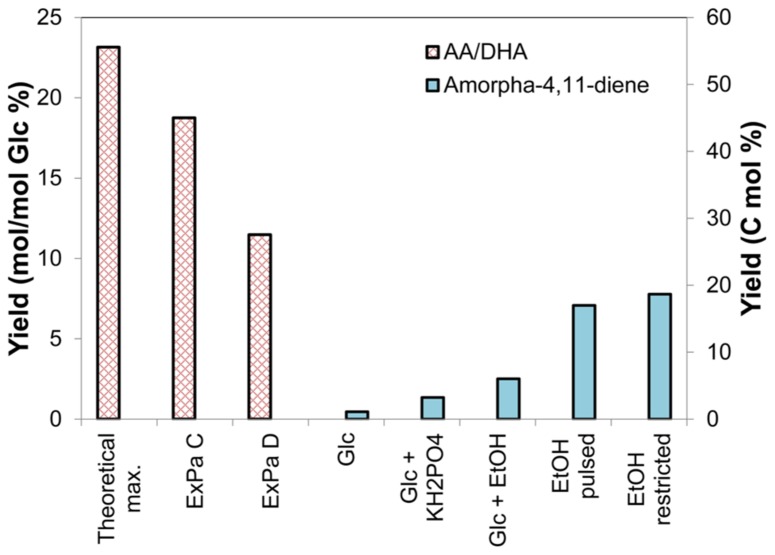
**Calculated (red bars) and previously reported (blue bars) yields of artemisinin precursors.** The theoretical maximum yield of the artemisinin precursors amorpha-4,11-diene, AA, or DHA is 22 mol/100 mol glucose (Glc) (left axis) or 55 C mol % (right axis). One ExPa (ExPa C, with ME2 active), yields 18 mol DHA/100 mol glucose), with concomitant biomass production (3 mol/100 mol glucose). Here, ME2 supplies reductant to meet the NADPH requirement. Another ExPa (ExPa D, with an active oxidative PPP), yields 11 mol DHA/100 mol glucose, with concomitant biomass production (6 mol/100 mol glucose). Here, the oxidative PPP supplies reductant to meet the NADPH requirement. **Figure 3** depicts flux distributions through these ExPas. The yields of artemisinin precursors in the pioneering microbial production studies (Ro et al., 2006; Zhang et al., 2011) were relatively low; however, subsequent work obtained further improvement in amorpha-4,11-diene and AA yield by increasing flux through the MVA pathway and varying feed composition. Recently, Westfall et al. (2012) achieved drastic improvement in amorpha-4,11-diene yield of close to 20 C mol % by using an optimized yeast strain with a mixed feed consisting of ethanol (EtOH) and Glc. They achieved yields of 1.1 C mol % with glucose as carbon source, which increased to 3.2 C mol % by using KH_2_PO_4_ with the glucose feed, to 6.0 C mol % by using glucose and EtOH mixed feed. The highest yields obtained were 17.0 and 18.7 C mol % with a restricted EtOH feed.

The AA route for artemisinin production has recently seen impressive scientific success and commercialization (Hale et al., 2007; Chandran et al., 2008; Lenihan et al., 2008; Westfall et al., 2012; Paddon et al., 2013). The titers and yields of artemisinin or its precursors have been improved substantially by optimization of downstream metabolic pathways as well as process development. The work of Keasling and coworkers improved FPP production, and thereby AA titers, in the initial AA-synthesizing strains (Ro et al., 2006) by ingeniously combining several approaches. These included (i) overexpression of *upc2-1,* a transcription factor involved in the regulation of sterol production, to increase flux through the MVA pathway; (ii) downregulation of squalene synthase (*ERG9*)*,* which diverts FPP away from DHA production to sterol production; and (iii) overexpression of 3-hydroxy-3-methylglutaryl (HMG)-CoA reductase (*HMGR*) to enhance conversion of HMG-CoA to MVA. By employing these strategies in concert, Keasling and colleagues were able to improve the titer of amorpha-4,11-diene, a precursor of the artemisinin pathway (**Figure 1**), by more than 15-fold over the original AA-synthesizing strain (Ro et al., 2006). Further work (Lenihan et al., 2008) improved AA titers of these strains through process optimization of fermentation parameters. Recently, Keasling and colleagues employed the strategy of overexpressing all enzymes of the MVA pathway together with repression of *ERG9* to achieve very high titers and yields of amorpha-4,11-diene and AA (Westfall et al., 2012). While all previous attempts employed galactose or glucose as the sole carbon source, Westfall et al. (2012) fed ethanol as a second carbon source and obtained yields of amorpha-4,11-diene higher than 18 C mol % (**Figure 2**). More recently, Paddon et al. (2013) improved AA titers by more than 15-fold to 25 g L^-1^. Coupled with their development of a scalable chemical process for conversion of AA to artemisinin, this work is a major breakthrough in the commercial production of artemisinin.

The approaches described above have improved AA yields almost exclusively by manipulating flux through downstream pathways, specifically the MVA and sterol biosynthesis pathways. However, another powerful approach to enhance the production of artemisinin precursors would be to engineer upstream (primary and central carbon) metabolism. Several computational and experimental tools such as extreme pathway (ExPa) analysis (Wiback and Palsson, 2002; Llaneras and Picó, 2010), flux balance analysis (FBA; Orth et al., 2010), minimization of metabolic adjustment (MOMA) analysis (Segrè et al., 2002) as well as ^13^C metabolic flux analysis (^13^C MFA; e.g., Feng et al., 2010; Papini et al., 2012; Ahn and Antoniewicz, 2013; Nargund and Sriram, 2013) are now available to dissect metabolism, derive insight and propose metabolic engineering strategies. Because primary metabolic pathways supply substantial amounts of carbon and reductant to downstream pathways such as MVA and sesquiterpene synthesis, such strategies may identify non-trivial metabolic engineering strategies and thus play a very significant role in improving flux to AA or DHA. There have been relatively few reports of computationally inferred genetic engineering strategies in upstream metabolism for improving microbial yields of intermediary or secondary metabolites. Studies that have successfully accomplished this include those focusing on the production of L-valine (Park et al., 2007), lycopene (Alper et al., 2005), and C_14_–C_16_ fatty acids (Ranganathan et al., 2012) by *Escherichia coli*. In one study directed at secondary metabolite production by yeast, Asadollahi et al. (2009) identified genetic interventions toward increased production of sesquiterpenes synthesized via the MVA pathway, by *in silico* analyses of a genome-scale model of yeast metabolism. They predicted that knocking out glutamate dehydrogenase (*GDH1*), whose product assimilates nitrogen at the expense of NADPH, will result in a 10-fold increase in the production of the sesquiterpene cubebol. Mechanistically, this knockout was predicted to shunt carbon via an alternative reaction that consumes NADH instead of NADPH, thus improving NADPH availability to the MVA pathway and sesquiterpene synthesis. Indeed, on *in vivo* implementation, the *GDH1* knockout strategy led to a significant (~twofold) increase in the titer of cubebol.

Because modeling approaches are immensely useful in synthetic biology (Zheng and Sriram, 2010), this article investigates strategies for improving DHA yields in engineered yeast by employing a variety of computation-assisted methodologies. These include ExPa analysis, ^13^C MFA, FBA, and MOMA analysis. ExPa and elementary flux mode analysis enable detailed investigation of the flux distribution space (phenotypic space) of a metabolic network (Schilling et al., 2000) to obtain insights on the network’s capabilities and limitations. Such analyses can suggest genetic intervention strategies to effect a desired outcome from the network Becker et al. (2011). ^13^C MFA is a powerful methodology currently used for estimating intracellular fluxes. In this methodology, isotope labeling signatures obtained from feeding a mixture of ^13^C and ^12^C carbon sources to an organism, in conjunction with extracellular flux measurements, are used to evaluate intracellular flux distributions (e.g., Wiechert, 2001; Sriram et al., 2004, 2008; Murphy et al., 2013). FBA, a complementary approach to ^13^C MFA, optimizes a metabolic objective to estimate or predict flux distribution seven at the genome-scale (Grafahrend-Belau et al., 2009; Orth et al., 2010). In genome-scale FBA, an organism’s inventory of metabolic reactions is used to assemble a stoichiometric matrix. Thermodynamic irreversibility constraints as well as measurements of a few extracellular fluxes such as substrate uptake and product secretion are used to constrain the null space of this matrix, thus generating a phenotypic space of feasible flux distributions. An objective function such as maximization of biomass production is then employed to isolate a particular solution within the feasible solution space. Several extensions of the FBA approach enable prediction of changes in flux distributions due to genetic interventions, including OptKnock (Burgard et al., 2003), OptStrain (Pharkya et al., 2004), OptForce (Ranganathan et al., 2010), and MOMA (Segrè et al., 2002). MOMA predicts flux alterations resulting from gene knockouts, and thereby enables identification of advantageous knockouts. It is based on the premise that gene knockouts restrict the wild type phenotypic space of an organism, and that organisms respond by minimally adjusting their wild type flux distribution to make it lie within the restricted phenotypic space (Segrè et al., 2002). This contrasts with FBA, which assumes that organisms respond by finding a new optimum of their objective function within the restricted phenotypic space.

To our knowledge, this is the first study utilizing a variety of computational and experimental pathway analysis techniques to identify metabolic engineering targets for enhanced production of artemisinin precursors in yeast. Together, our analyses elucidate upstream bottlenecks in DHA synthesis and suggest non-trivial genetic intervention strategies for pushing carbon toward the DHA pathway and thereby improving DHA yield.

## MATERIALS AND METHODS

### ExPa ANALYSIS

For ExPa analysis, we constructed a metabolic model of yeast engineered to synthesize DHA. This model consisted of central carbon metabolic pathways native to yeast including glycolysis, pentose phosphate pathway (PPP), tricarboxylic acid (TCA) cycle, MVA pathway, pathways for synthesis of biomass components and a consolidated reaction encompassing the pathway from MVA to DHA. Altogether, this model contained 63 reactions and 51 metabolites. To determine ExPas, we analyzed this model with the *expa* program from Bell and Palsson (2004). The resulting ExPas are tabulated in Data Sheet 1 in Supplementary Material.

### CELL CULTURE, STRAIN CHARACTERIZATION, AND METABOLIC MEASUREMENTS

#### Yeast cell culture in microplates

We used three *S. cerevisiae* strains in this study (Fy_AA_, Fy_DHA_, and Fy_0_; Zhang et al., 2008), all of which were designer yeast strains carrying lysine and methionine auxotrophies. Strain Fy was engineered with genes for the synthesis of AA, specifically *A. annua* farnesyl diphosphate synthase (*FDS*), amorpha-4,11-diene synthase (*ADS*), cytochrome P450 monooxygenase (*CYP71AV1*), and *A. annua* cytochrome P450 reductase (*CPR*). Strain Fy_DHA_ was engineered with genes for the synthesis of DHA, specifically *FDS*, *ADS*, *CYP71AV1*, *CPR*, and *A. annua* double bond reductase (*AaDBR2*). **Figure 1** depicts the reactions catalyzed by the products of these genes. Strain Fy_0_ was an empty vector control. Further genetic engineering details are provided in the supplementary material of Zhang et al. (2008). We grew the strains in synthetic defined base yeast minimal medium (Clontech Laboratories, Mountain View, CA, USA) supplemented with trace amounts of lysine and methionine due to the auxotrophies in the strains. This medium was supplemented with 2% (w/v) glucose or galactose as carbon source. All growth and ^13^C MFA experiments were performed on 2 mL batch cultures in deep-well microplates in a Biotek Synergy HT microplate reader (Biotek Instruments, Winooski, VT, USA) at 30°C with continuous shaking. To induce DHA or AA production, cultures grew for 45–55 h on this medium supplemented with 2% (w/v) galactose as the carbon source. For isotope labeling studies, we used either 20% U-^13^C galactose or 100% 1-^13^C galactose in parallel labeling experiments. To harvest cells we centrifuged the cell suspensions, separated the supernatant and immediately froze the pellets in liquid nitrogen to arrest metabolism. The pellets and supernatants were lyophilized and stored at -80°C until further analysis.

#### Strain characterization

Expression of recombinant genes in the engineered strains was confirmed by RT-PCR, using primers from Zhang et al. (2008). *A. annua actin1* was used as a negative control, whereas the yeast *ACT1* and *TAF10* were used as positive controls (Teste et al., 2009). Primers (sequences are listed in Data Sheet 2 in Supplementary Material) were obtained from Integrated DNA Technologies (Coralville, IA, USA).

Cell growth rates were determined via online optical density measurements on the Biotek microplate reader. Measurements for glucose, ethanol, and glycerol were performed by analyzing the supernatant with a YSI 2700 Select metabolite analyzer (YSI Life Sciences, Yellow Springs, OH, USA), using a measurement kit appropriate for each metabolite. These measurements are listed in Data Sheet 2 in Supplementary Material. AA and DHA were extracted by using protocols adapted from Zhang et al. (2008) and quantified by gas-chromatography-mass spectrometry (GC-MS) of their TMS derivatives, using methyl stearate as an internal standard.

#### Extraction and analysis of proteinogenic amino acids for ^13^C isotopomer measurements

Proteinogenic amino acids in cell pellets were obtained by vacuum-hydrolyzing the pellets with 6N hydrochloric acid (Thermo Scientific, Rockford, IL, USA). The hydrolysate was mixed with a known amount of norleucine as an internal standard and derivatized by adding 100 μL *N*-(tert-butyldimethylsilyl)-*N*-methyltrifluoroacetamide (MTBSTFA; Thermo Scientific) in 100 μL dimethylformamide (Thermo Scientific) and heating at 70°C for 1 h. GC-MS analysis of 1 μL of this derivatized mixture was performed using previously published instrument methods (Sriram et al., 2008) on a Varian 300MS quadrupole GC-MS unit (Bruker, Billerica, MA, USA) equipped with a VF5-ms column of dimensions 0.25 mm × 30 m × 0.25 μm and capable of detecting ions by electron ionization. Quantification of amino acids in the biomass was performed by comparing the chromatographic peak of each amino acid with that of the norleucine internal standard. Isotopomer abundances obtained from the mass spectrum of each amino acid were processed to filter out natural abundances of elements other than metabolic carbon, using a previously developed in-house MATLAB program, whose accuracy has been verified by using amino acid isotopomer mixtures of known isotopomeric composition (Sriram et al., 2008). Isotopomer abundances for the three strains are reported in Data Sheet 2 in Supplementary Material.

#### Reproducibility and statistics

All experiments were performed with two or three biological replicates, and statistical significance was determined by calculating *p*-values through a Student’s *t*-test.

#### Evaluation of metabolic fluxes from ^13^C isotopomer data

We evaluated fluxes from the ^13^C mass isotopomer data and extracellular flux measurements by using our flux evaluation program NMR2Flux+ (Sriram et al., 2004, 2008) to fit this data to a steady state flux-isotopomer model. This model consisted of yeast central carbon metabolic pathways, including glycolysis, PPP, TCA cycle, and anaplerotic pathways. For the retrobiosynthetic reconstruction of metabolic precursor mass isotopomer distributions (MIDs) from the amino acid MIDs (e.g., pyruvate from alanine or oxaloacetate from aspartic acid), we used standard pathways from the precursors to the amino acids (Szyperski, 1995, 1998). These pathways are universally conserved across life (Romano and Conway, 1996). We used standard models for the biosynthesis of amino acids from the intermediates of these pathways (e.g., Szyperski, 1995). We found that labeling of the proteinogenic amino acids except methionine and lysine was very close to 20% for cells grown on 20% U-^13^C galactose, which indicates attainment of isotopic steady state (data not shown). However, the metabolism is still at pseudo-steady state due to the batch culture. Statistical analysis was performed by the NMR2Flux+ program, using a bootstrap Monte Carlo simulation to execute 100 runs of the flux evaluation by perturbing measurements by their known standard error, as previously described (Sriram et al., 2004, 2008). Evaluated fluxes for the three strains are reported in Data Sheet 2 in Supplementary Material.

### FBA AND MOMA ANALYSIS

We modified the iMM904 genome-scale model for *S. cerevisiae* (Mo et al., 2009) by appending it with the reactions necessary to synthesize DHA from FPP. We constrained this model with the measured extracellular uptake fluxes (glucose or galactose) as well as intracellular fluxes estimated from the ^13^C isotopomer data. The open-source COBRA toolbox (Schellenberger et al., 2011) was used to perform FBA and MOMA analysis on the model. For FBA, we used maximization of biomass growth as the objective function. We performed MOMA to assess the effects of all possible single-gene knockouts on DHA production. For this, we used an in-house MATLAB script (available on request) to automate certain repetitive steps such as recurring calls to COBRA. The non-lethal single-gene knockouts that significantly increased DHA yields were used in a second iteration of the MOMA analysis to identify double-gene knockouts that enhanced DHA production. The results of this analysis are available in Data Sheet 3 in Supplementary Material.

## RESULTS AND DISCUSSION

The currently available synthesis routes for DHA (or AA) use galactose or glucose as carbon sources (Ro et al., 2006; Teoh et al., 2006; Zhang et al., 2008; Paddon et al., 2013). Strategizing how to produce DHA from these substrates at high yield is a complex problem, solving which requires answering the following questions. (i) What is the theoretical maximal yield of DHA on glucose or galactose? (ii) Is this yield limited by the availability of reductant cofactors? (iii) Which configuration of pathways and fluxes favors a high yield? (iv) Do current DHA-synthesizing strains operate near or distantly from such a configuration? (v) Which minimal sets of genetic interventions can drastically improve the yield of a low DHA-producing strain? Below, we describe our computational analyses (ExPa analysis and MOMA analysis) and experimental investigations (^13^C MFA) toward answering these questions.

### THEORETICAL MAXIMAL YIELD OF DHA OR AA ON GLUCOSE

The value of the maximal yield of DHA on glucose (or a similar sugar such as galactose) depends on whether the reductant cofactors NADH and NADPH are considered in the analysis, and whether a distinction is made between the cofactors NADH and NADPH. A cofactor-free theoretical yield calculation reveals that yeast can produce a maximum yield of 22 mol DHA/100 mol glucose, or 1 mol DHA per 4.5 mol glucose (**Figure 2**). Metabolically, this would proceed as follows. Glycolysis would convert 4.5 mol glucose to 9 mol acetyl-CoA, which the MVA pathway would condense to form 3 mol dimethylallyl diphosphate (DMAPP). DMAPP would further condense to form FPP in a molar proportion of 3 DMAPP:1 FPP. FPP would then be converted to amorphadiene and ultimately to DHA in a 1:1 molar proportion (Bertea et al., 2005). This DHA yield is equivalent to 55 C mol %, wherein the 45 C mol % carbon loss is due to the release of CO_2_ in multiple pathway steps. This calculation and yield value also apply to AA production from glucose or galactose.

The fact that artemisinin, DHA and AA are significantly reduced compounds necessitates the consideration of reductant (cofactor) demand and supply in this yield calculation. In the upstream section of the pathway described above, the conversion of 4.5 mol glucose to 9 mol acetyl-CoA supplies 18 mol NADH. In the downstream section, 6 mol NADPH is required for the conversion of 9 mol acetyl-CoA to 3 mol DMAPP. Therefore, if no distinction is made between the NADH and NADPH, this pathway is self-sufficient in reductant because the demand of 6 mol NADPH is met by a supply of 18 mol NADH.

The interchangeability of NADH and NADPH invoked in the above analysis is contingent upon the availability of a transhydrogenase enzyme that converts NADH to NADPH (Stephanopoulos et al., 1998). However, there is no evidence for a transhydrogenase in yeast (Bruinenberg et al., 1983; Nissen et al., 2001). Therefore, other NADPH-supplying pathways will have to be recruited to make DHA production feasible. This complicates the theoretical yield calculation for three reasons. Firstly, multiple pathways including the oxidative PPP and anaplerotic cycles can supply NADPH either by themselves or in combination. Secondly some of these pathways, such as the oxidative PPP, supply NADPH at the expense of carbon, so that recruiting them for NADPH provision can compromise the maximal yield of DHA. Thirdly, the practical necessity of concurrently producing biomass with DHA will obviously reduce the maximal yield of DHA. To analyze this complex set of possibilities and delineate all possible pathway configurations for DHA synthesis with or without cofactor requirement, we performed ExPa analysis of the underlying metabolic network.

### ExPa ANALYSIS OF DHA SYNTHESIS NETWORK

**FIGURE 3:**
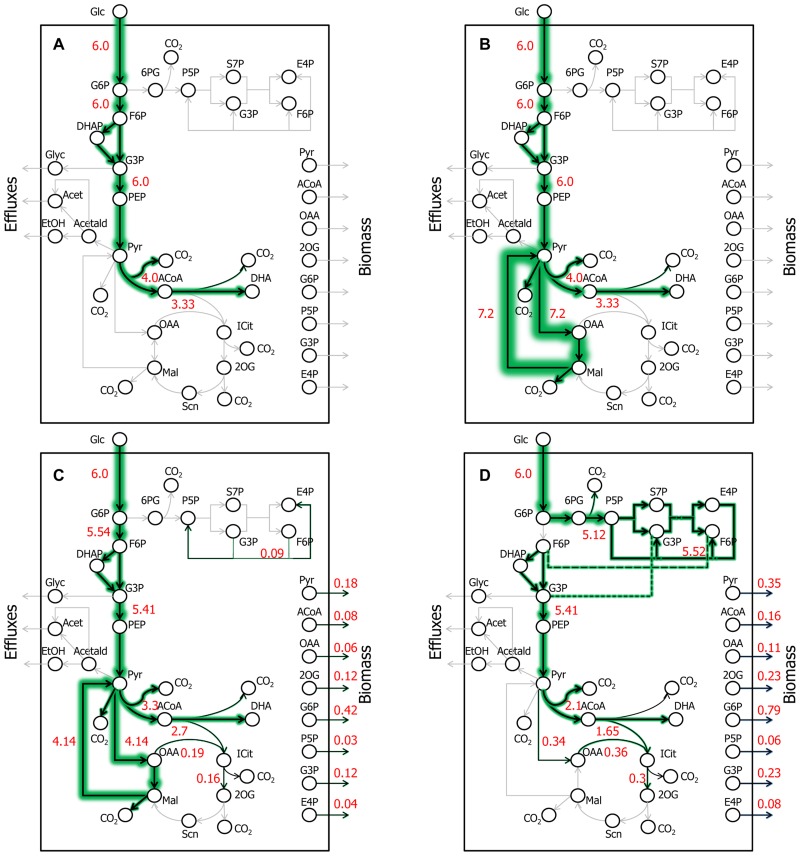
**Key DHA- or AA-synthesizing ExPas in yeast.** Four ExPas that synthesize DHA (or AA) and/or biomass are shown. Each ExPa is depicted on a network diagram by highlighting the reactions active in it. The intensities of green glows around arrows are proportional to the fluxes of the corresponding reactions; numerals alongside the arrows indicate flux values. **(A)** ExPa A, producing the theoretical maximum DHA yield of 22 mol/100 mol glucose, without considering cofactor requirement or availability. There is no biomass production and all possible carbon is diverted toward DHA synthesis. **(B)** ExPa B, producing the theoretical maximum DHA yield of 22 mol/100 mol glucose, by accounting for cofactor requirement and availability. There is no biomass production. Here, the NADPH requirement is met by a pyruvate ≫ oxaloacetate ≫ malate ≫ pyruvate shuttle via ME2. **(C)** ExPa C, producing a high DHA yield of 18 mol/100 mol glucose, with concomitant biomass production of 3 mol/100 mol glucose. ME2 supplies reductant to meet the NADPH requirement. **(D)** ExPa D, producing substantial DHA yield of 11 mol/100 mol glucose, with concomitant biomass production of 6 mol/100 mol glucose. Here, the oxidative PPP supplies reductant to meet the NADPH requirement.

Colloquially, an ExPa represents one way of “walking through a metabolic network.” Mathematically, ExPas are the edges of the polyhedral cones that represent the hyperdimensional phenotypic space that a metabolic network can occupy (Bell and Palsson, 2004; Palsson, 2006). ExPa analysis has previously been applied to a variety of systems on different scales from small scale networks of red blood cells to genome-scale pathway analysis of the influenza virus to elucidate important metabolic properties of the systems (Schilling and Palsson, 2000; Wiback and Palsson, 2002). Amongst the three types of ExPas (types I, II, and III) classified by Palsson (2006), we are interested in types I and III. Type I ExPas effect the conversion of a substrate or substrates to a product or products along with the transport of the substrates and products across the cell membrane. Type III ExPas involve intracellular cycles with no transport across the cell membrane. To perform ExPa analysis, we constructed a simplified (less than genome-scale) metabolic model of yeast capable of producing both DHA and biomass (Data Sheet 1 in Supplementary Material). This model includes 63 reactions including exchange reactions and 51 metabolites including cofactors. We then performed ExPa analysis on the model by (i) neglecting and (ii) considering cofactor requirements to gain insights about the capabilities of the network. **Table 1** presents a summary of ExPa families for both cases, and **Figure 3** depicts flux distributions through four selected ExPas. Of these, ExPa A was obtained from a cofactor-free model, and ExPas B, C, and D were obtained by considering cofactor requirements.

**Table 1:** Summary of ExPa families for a yeast strain synthesizing DHA from glucose, in the absence and presence of cofactor requirement.

	Cofactor-free			With cofactor requirement		
ExPa family^a^	# of ExPas	Maximal yields		# of ExPas	Maximal yields	
DHA production^b^	5(#1 to #5)	DHA:	22	238(#1 to #238)	DHA:	22
DHA and biomass production^c^	0	N/A		94(#239 to #332)	DHA:	18
					Biomass:	10
Biomass production	11(#6 to #16)	Biomass:	16	383(#333 to #715)	Biomass:	11
All carbon lost to CO_2_	10(#17 to #26)	CO_2_:	600	108(#716 to #823)	CO_2_:	600
Metabolite production via glycolysis	10(#27 to #36)	Glycerol:	200	83(#824 to #906)	Glycerol:	164
		Ethanol:	200		Ethanol:	200
Metabolite production via PPP	14(#37 to #50)	Glycerol:	167	161(#907 to #1067)	Glycerol:	137
		Ethanol:	167		Ethanol:	167
Type II ExPas	6(#51 to #56)	N/A		0	N/A	
Type III ExPas	18(#57 to #74)	N/A		19(#1068 to #1086)	N/A	

*ExPa analysis revealed maximal DHA yield of 22 mol/100 mol glucose in both cases. Simultaneous DHA and biomass production was possible only when cofactor requirements were considered. The numerals following the hashes “#” in the “# of ExPas” columns denote pathway numbers in Data Sheet 1 in Supplementary Materials. All yields are in mol/100 mol glucose.*

a ExPas are type I unless otherwise specified.See Palsson (2006) and text for definitions of ExPa types.

b NADPH corresponding to maximal DHA yield is supplied by the ME2 reaction; supply of NADPH by the PPP results in lower yields.

c This is the most important ExPa family, as it provides insights into strategies for improving overall DHA yield.

### COFACTOR-FREE ExPa ANALYSIS REVEALS IMPORTANT CLASSES OF PATHWAYS TOWARD DHA SYNTHESIS

A cofactor-free ExPa analysis of a network, although an incomplete depiction of the network, permits the delineation of major classes of metabolic routes through it. Such an analysis of the metabolic model in Data Sheet 1 in Supplementary Material****revealed 50 type I, 6 type II, and 18 type III ExPas (**Table 1** and Data Sheet 1 in Supplementary Material). Of these, only five ExPas synthesized DHA as their sole product, whereas the rest synthesized various secreted metabolites (e.g., glycerol, ethanol, and acetate), biomass or a combination of biomass and secreted metabolites. Interestingly, none of the biomass-producing ExPas concurrently synthesized DHA (**Table 1**). The DHA yield in the DHA-synthesizing ExPa A was equal to the previously calculated theoretical maximum of 22 mol DHA/100 mol glucose (**Figure 3A**).

### INCLUSION OF COFACTOR REQUIREMENT RECRUITS NADPH-MALIC ENZYME (ME2) OR THE OXIDATIVE PPP FOR NADPH PROVISION TO DHA SYNTHESIS PATHWAY

We repeated the foregoing ExPa analysis for a more realistic scenario that considered cofactor requirements and did not include a transhydrogenase to interconvert NADH and NADPH. This analysis resulted in 1086 ExPas with 1067 type I, 0 type II, and 19 type III ExPas (**Table 1** and Data Sheet 1 in Supplementary Material). Interestingly, the maximal DHA yield obtained from this analysis was identical to the theoretical maximal yield, indicating that some reactions in the network are able to supply the desired cofactors without shunting any carbon away from DHA production. For instance, one ExPa (ExPa B) gave a DHA yield of 22 mol/100 mol glucose by providing NADPH via ME2 with no concomitant biomass production (**Figure 3B**). Here, a pyruvate ≫ oxaloacetate ≫ malate ≫ pyruvate shuttle, with the malate ≫ pyruvate conversion catalyzed by ME2, meets the NADPH requirement of DHA synthesis. In all the 238 ExPas that exclusively synthesized DHA, the NADPH requirements were met either by a coupling of the pyruvate carboxylase and ME2 reactions or by operation of the oxidative PPP. In fact, the ME2 reaction for converting malate to pyruvate and the oxidative branch of the PPP are the only two means of producing NADPH in the network. ExPas that recruited the oxidative PPP to meet the NADPH requirement were associated with a DHA yield lower than the theoretical maximum, because the oxidative PPP loses significant carbon as CO_2_. Consequently, ME2 is preferable to the oxidative PPP as a source of NADPH for DHA synthesis. This makes ME2 an attractive metabolic engineering target.

Consideration of cofactor requirements resulted in an important set of 94 ExPas that simultaneously produced DHA and biomass (Data Sheet 1 in Supplementary Material and **Table 1**; see examples in **Figures 3C,D**). These pathways are important to our objective because an overall improvement in DHA yield requires the production of both DHA and biomass at high yields. However, concomitant synthesis of DHA and biomass involves many NADPH-consuming reactions with conflicting NADPH demands. The NADPH demands of DHA biosynthesis are explained earlier in this section; biomass synthesis requires NADPH for the conversion of central carbon metabolic precursors to proteinogenic amino acids and lipogenic fatty acids. Thus, an increase in DHA yield is accompanied by a decrease in biomass yield and vice versa. Two important ExPas that coupled DHA and biomass production are depicted in **Figures 3C,D**. ExPa C (**Figure 3C**) produced a high DHA yield of 18 mol/100 mol glucose, with concomitant biomass production of 3 mol/100 mol glucose. ME2 supplied reductant to meet the NADPH requirement. ExPa D (**Figure 3D**) produced substantial DHA yield of 11 mol/100 mol glucose, with concomitant biomass production of 6 mol/100 mol glucose. Here, the oxidative PPP supplied reductant to meet the NADPH requirement. Thus, the ExPa analysis provided insights on the capabilities of the DHA biosynthesis metabolic network, and identified ME2 and the oxidative PPP as two critical metabolic engineering targets.

However, ExPa analysis only identifies the modes of the network, as each ExPa is the edge of the realizable flux cone. Because a real flux distribution would be a linear combination of a number of ExPas, it will contain pathways that produce unneeded metabolites. Furthermore, ExPa analysis does not take in to account the carbon requirement for the *de novo* synthesis of cofactors and energy carriers such as ATP. In the absence of these processes that consume carbon, it overestimates production of biomass and other metabolites compared to experimental results. As a result, the actual improvements in yields would be lower than predicted by the ExPas analysis. To obtain more realistic predictions, we performed flux determination of the DHA-synthesizing strain experimentally using ^13^C MFA.

### ^13^C MFA AND EXTRACELLULAR MEASUREMENTS OF DHA-SYNTHESIZING STRAIN REVEAL NEGLIGIBLE PPP AND LOW TCA CYCLE FLUXES AS WELL AS SUBSTANTIAL FERMENTATIVE FLUXES

**FIGURE 4:**
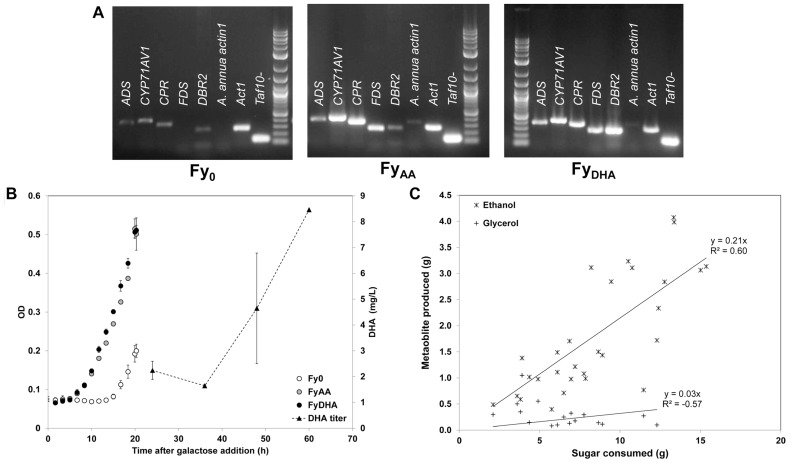
**Characterization of engineered yeast strains Fy_0_, Fy_AA_, and Fy_DHA_ used in this study.** **(A)** Gene expression assay of *ADS*, *CYP71AV1*, *CPR*, *FDS*, *DBR2*, a negative control (*A. annua actin1*) and two positive controls (*ACT1* and *TAF10*). This assay was performed in duplicate at multiple time points; only one replicate is shown. This assay showed that as expected, strain Fy_AA_ expresses *ADS*, *CYP71AV1*, *CPR*, and *FDS*, whereas strain Fy_DHA_ expresses these genes and *DBR2*. **(B)** Cell growth curves (left axis) and DHA titer for Fy_DHA_ strain (right axis): white circles, Fy_0_; gray circles, Fy_AA_; black circles, Fy_DHA_; black triangles, DHA titer of Fy_DHA_. **(C)** Yield calculations of ethanol (asterisks) and glycerol (plus signs) on glucose and galactose for all three strains.

We performed comparative ^13^C MFA of the three yeast strains Fy_0_, Fy_AA_, and Fy_DHA_. First, we used RT-PCR to verify that these strains correctly express the recombinant genes engineered into them (**Figure 4A**). We also verified the strains produce the secondary metabolites expected of them: Fy_AA_ produced AA but not DHA, Fy_DHA_ produced both DHA and AA, whereas Fy_0_ produced neither compound (data not shown). **Figure 4B** depicts transient DHA production by Fy_DHA_. Extracellular flux measurements on these strains (**Figure 4C**) showed that ~50% of the consumed galactose was directed toward ethanol, 1% toward glycerol and less than 10% toward biomass. Thus, despite aerobic conditions, the strains exhibited substantial fermentative metabolism. Therefore, an initial strategy for improving DHA yields should be the deletion of the ethanol fermentation pathway, which can be expected to double the carbon available for DHA and biomass production.

**FIGURE 5:**
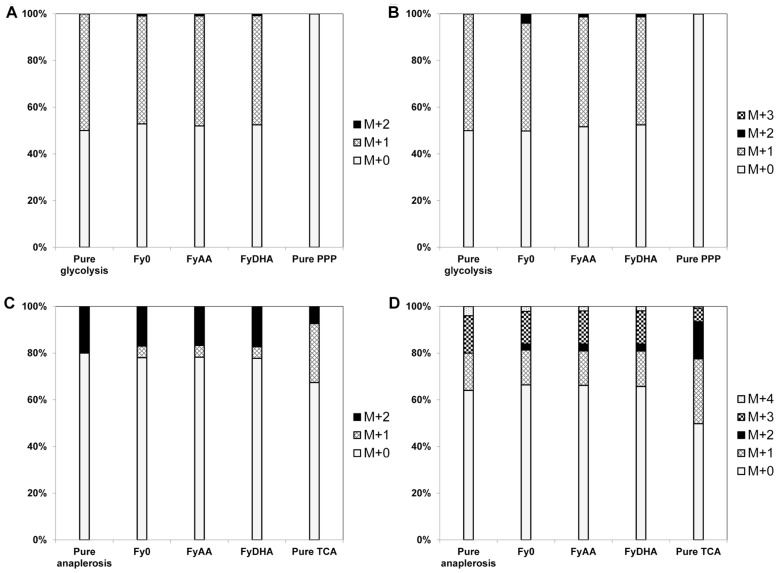
**^13^C labeling patterns of proteinogenic amino acids evince negligible PPP flux and lowTCA cycle flux in all three yeast strains.** **(A)** Ala[23] fragment, **(B)** Ala[123] fragment, **(C)** Asp[12] fragment **(D)** Asp[1234] fragment. The observed MIDs of the Ala fragments of all three strains closely resembled MIDs simulated by assuming that all carbon is processed by glycolysis (“pure glycolysis” bar), and were substantially different from MIDs simulated by assuming that all carbon passed through the PPP. This evidences negligible PPP flux in all three strains. The observed MIDs of both the Asp fragments more closely resembled simulated MIDs corresponding to the synthesis of oxaloacetate through anaplerosis than those corresponding to oxaloacetate synthesis through the TCA cycle. This suggests lowTCA cycle flux in all three strains.

**FIGURE 6:**
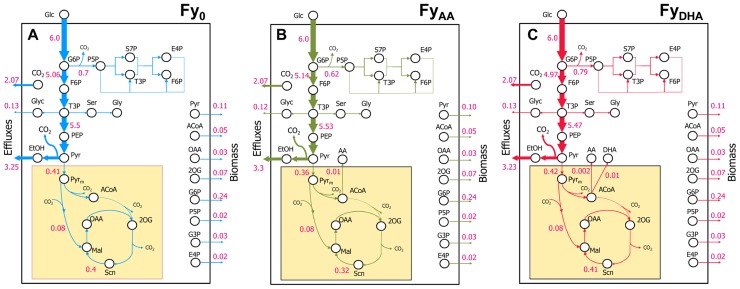
**Flux distributions obtained by ^13^C MFA for the three engineered yeast strains.** **(A)** Fy_0_; **(B)** Fy_AA_; **(C)** Fy_DHA_. All three strains show similar flux distributions with a substantial fraction of glucose carbon shunted to ethanol and glycerol production. All strains exhibited large fluxes through glycolysis and negligible or low fluxes through both the oxidative PPP and the TCA cycle.

For a more profound understanding of the metabolic behavior of these strains, we performed ^13^C MFA on them. **Figure 5** depicts key isotopomer trends that emerged from the ^13^C labeling experiments. Surprisingly, we did not observe any major significant differences in the amino acid isotopomer abundances between the three strains (Data Sheet 2 in Supplementary Material). This was confirmed by a principal component analysis of the isotopomer abundance data, in which all three strains clustered quite close together, although the Fy_AA_ and Fy_DHA_ and strains were slightly closer to each other than each was to the control strain Fy_0_ (data not shown). **Figure 6** presents the flux maps obtained from ^1^^3^C MFA, using the metabolic model listed in Data Sheet 2 in Supplementary Material. This model did not consider cofactors. NADPH and other cofactors are often consumed in processes such as scavenging reductive oxygen species, making it difficult to estimate fluxes in real cellular scenarios. Because ^13^C MFA obtains significant flux information from isotopomer abundance data, it is a powerful method of estimating fluxes independent of cofactors.

^13^C MFA revealed that almost all the galactose entering the cell was metabolized via glycolysis with ~10% of it passing through the oxidative PPP (**Figure 6**). This is clear from the MIDs of the fragment of alanine in the 100% 1-^13^C galactose labeling experiment (**Figures 5A,B** and Data Sheet 2 in Supplementary Material). The [123] fragment of pyruvate (and of alanine, which is derived from it) is synthesized from the [321] and [456] fragments of galactose. The oxidative PPP results in the loss of the C-1 carbon of galactose. Thus, alanine synthesized from carbon that passed through the oxidative PPP should exhibit a [123] fragment with the MID {*m*+0: 100%; *m*+1: 0%; *m*+2: 0%; *m*+3: 0%}. Conversely, alanine synthesized from carbon that passed through glycolysis should exhibit a [123] fragment with the MID {*m*+0: 50%; *m*+1: 50%; *m*+2: 0%; *m*+3: 0%}. In our 100% 1-^13^C galactose labeling experiment, we observed an MID of {*m*+0: 52%; *m*+1: 46%; *m*+2: 1%; *m*+3: 0%} for the alanine [123] fragment (**Figure 5B**), indicating that the oxidative PPP was nearly inactive. Data from other amino acid fragments in this experiment as well as the 20% U-^13^C galactose labeling experiment also concur with this result. Because the oxidative PPP generates two NADPH molecules for every molecule of glucose-6-phosphate metabolized through it and is featured in many DHA-synthesizing ExPas identified above, amplifying flux through it is a key metabolic engineering strategy toward improving DHA yield. A possible candidate gene for overexpression is glucose-6-phosphate dehydrogenase *G6PDH2*, which is known to be the rate-limiting enzyme in this pathway in *S. cerevisiae* (Kwon et al., 2006).

Furthermore, the isotopomer data revealed that the TCA cycle carried lower flux than expected under aerobic conditions (**Figures 5C,D**). This is evident from the MIDs of the aspartic acid fragment [1234] {*m*+0: 66%; *m*+1: 15%; *m*+2: 3%; *m*+3: 14%; *m*+4: 2%} and alanine fragment [123] {*m*+0: 78%; *m*+1: 3%; *m*+2: 1%; *m*+3: 18%}from the 20% U-^13^C galactose labeling experiment (**Figure 5D** and Data Sheet 2 in Supplementary Material). Assuming that the ^13^C enrichment of intracellular CO_2_ is approximately the same as that of the supplied galactose, synthesis of the fragment [1234] of the TCA cycle intermediate oxaloacetate, and thereby of aspartic acid, purely through the anaplerotic reaction pyruvate + CO_2_ ≫ oxaloacetate should result in the MID {*m*+0: 64%; *m*+1: 16%; *m*+2: 0%; *m*+3: 16%; *m*+4: 4%} for the aspartic acid [1234] fragment. Synthesis of oxaloacetate through the TCA cycle itself should result in a very different MID because of the carbon atom rearrangements in that pathway (**Figure 5D**). The closeness of the aspartic acid [1234] fragment MID to that predicted through pure anaplerotic synthesis indicates that the TCA cycle has very little flux relative to the anaplerotic reactions or glycolysis (**Figure 6**).

### COMPARISON OF ^13^C MFA AND ExPa FLUX DISTRIBUTIONS IDENTIFIES FURTHER METABOLIC ENGINEERING TARGETS

The selection of a preferred DHA-producing ExPa from amongst the 1000+ ExPas elucidated by us would depend on the ease with which the Fy_DHA_ strain can be engineered to emulate the ExPa. Therefore, as a step to identify gene targets for improved DHA production, we compared the flux distribution of strain Fy_DHA_ and the desired DHA- and biomass-synthesizing ExPas C and D from **Figures 3C,D** (**Table 2**). Because these ExPas are optimal DHA- and biomass-producing modes, emulating them should result in an improvement of overall DHA yield. Gene overexpressions necessary for Fy_DHA_ to emulate ExPa C include pyruvate dehydrogenase, ME2 and pyruvate carboxylase. Genetic interventions necessary to emulate ExPa D include overexpression of pyruvate dehydrogenase, glucose-6-phosphate dehydrogenase, transaldolase, and transketolase as well as knockdown of glucose-6-phosphate isomerase (**Table 2**). Common to both ExPas is pyruvate dehydrogenase, which catalyzes the pyruvate ≫ acetyl-CoA reaction. The rationale for overexpressing it is that the DHA-synthesizing strain has a low flux from pyruvate to acetyl-CoA, which would limit the carbon available for DHA production, as acetyl-CoA is the precursor for the MVA pathway. A strong alternative to pyruvate dehydrogenase would be acetyl-CoA synthetase, which plays a role in the pyruvate ≫ acetaldehyde ≫ acetate ≫ acetyl-CoA route that is reported to be a major source of acetyl-CoA in *S. cerevisiae* (Hynes and Murray, 2010). Another alternative may be ATP citrate lyase, which produces acetyl-CoA from citrate. However, this pathway may not be a major source of cytosolic acetyl-CoA in *S. cerevisiae* (Hynes and Murray, 2010).

**Table 2:** Genetic engineering targets determined by comparing ExPa analysis with ^13^C MFA.

	ExPa C	ExPa D
DHA yield	0.18	0.11
Biomass yield	0.03	0.06
NADPH supplied by	ME2	PPP
Gene overexpression in strain Fy_DHA_ necessary to emulate this ExPa	1. *YBR221C, YER178W, YFL018C, YGR193C, YNL071W* (pyruvate dehydrogenase)	1. *YBR221C, YER178W, YFL018C, YGR193C, YNL071W* (pyruvate dehydrogenase)
	2. *YKL029C* (NADPH-malic enzyme ME2)	2. *YNL241C* (glucose-6-phosphate dehydrogenase)
	3. *YBR218C, YGL062W* (pyruvate carboxylase)	3. *YGR043C, YLR354C* (transaldolase in PPP)
		4. *YBR117C, YPR074C* (transketolase in PPP)
Gene knockdown in strain Fy_DHA_ necessary to emulate this ExPa	1. *YBR145W, YGL256W, YOL086C* (alcohol dehydrogenase)	1. *YBR145W, YGL256W, YOL086C*(alcohol dehydrogenase)
		2. *YBR196C* (glucose-6-phosphate isomerase)

*The characteristics of two key ExPas with reasonable DHA and biomass yields are shown, with the corresponding genetic engineering targets to emulate these ExPas in Fy_DHA_. Common targets include eliminating ethanol biosynthesis by deleting alcohol dehydrogenase and increasing flux from pyruvate to acetyl-CoA by overexpressing pyruvate dehydrogenase.*

Other gene targets in **Table 2** belong to two categories: one involves improving flux through the PPP (overexpression of glucose-6-phosphate dehydrogenase, transketolase, transaldolase) and reducing the glycolytic flux (knockdown of glucose-6-phosphate isomerase), whereas the other involves increasing flux through the malate ≫ pyruvate reaction catalyzed by ME2. Both of these are complementary approaches that look to improve NADPH availability for DHA and biomass production. It should be noted that there is a likelihood of some ME2 flux in the yeast strains of this study. In the model we used for ^13^C MFA, all anaplerotic flux was consolidated into a single bidirectional reaction maef, because our isotopomer measurements could not resolve the different possibilities for this flux: phosphoenolpyruvate carboxykinase, phosphoenolpyruvate carboxylase, and pyruvate carboxylase (phosphoenolpyruvate or pyruvate + CO_2_ ≫ oxaloacetate) as well as NADH-dependent and NADPH-malic enzyme (pyruvate + CO_2_ ≪ ≫ malate). The flux distributions returned by ^13^C MFA included a low net flux (0.02) but substantial reversibility (0.97–0.98) for this consolidated anaplerotic flux, translating to forward (0.78) and reverse (0.76) fluxes that were significant, yet an order of magnitude smaller than the glycolytic flux (~5.0; Data Sheet 2 in Supplementary Material and **Figure 6**). ME2 may contribute to this flux, although this contribution remains unresolved. However, a comparison with ExPa B or C (**Figures 3B,C**) shows that even a 100% contribution by ME2 to this flux is insufficient to meet NADPH demand.

### FBA AND MOMA ANALYSIS OF THE MODIFIED iMM904 GENOME-SCALE MODEL REVEAL MULTIPLE GENETIC INTERVENTION STRATEGIES FOR IMPROVING DHA YIELDS

Flux balance analysis, a complementary approach to ^13^C MFA, estimates or predicts fluxes using linear programming techniques. It is particularly useful for analysis of metabolism at the genome-scale. MOMA is an extension of the FBA approach that identifies non-trivial gene knockouts toward optimizing a metabolic objective, such as the yield of a product. Its use toward determining gene targets has been reported in multiple successful studies (Alper et al., 2005; Park et al., 2007; Asadollahi et al., 2009; Ranganathan et al., 2012). We performed genome-scale FBA and MOMA analysis by using a modified iMM904 model (Mo et al., 2009). First, we used the fluxes obtained from ^1^^3^C MFA of the Fy_DHA_ strain for constraining fluxes in the iMM904 model, and then used FBA to predict a flux distribution for this strain. Then, we used MOMA analysis to simulate fluxes in single- and double-gene knockouts of the Fy_DHA_ strain to study the effects of these knockouts on DHA yields.

We initially observed that even with double-gene knockouts, DHA yields did not improve significantly over those of Fy_DHA_. This is because MOMA minimizes flux changes between the knocked out and control strains, and the low fluxes of NADPH-supplying reactions in the Fy_DHA_ strain prevented MOMA from identifying knockouts for improving DHA yield. Consequently, we modified the flux distribution of Fy_DHA_ *in silico* such that it had equal fluxes through PPP and glycolysis, but its DHA yield was the same as the measured yield of Fy_DHA_. We performed subsequent MOMA analysis on this new baseline strain Fy_DHA_’. Subsequent MOMA analysis found 401 single-gene knockouts with significantly increased DHA flux compared to Fy_DHA_’. We then used the single-gene knockouts associated with the highest DHA yields to identify double-gene knockouts that would potentially exhibit even higher DHA yields. The MOMA analysis predicted substantially improved DHA yields in these knockouts (Data Sheet 3 in Supplementary Material). In many cases, the increase in DHA yield was accompanied by an expected decrease in biomass yield, because knocking out genes can lead to suboptimal growth as the products of the knocked out genes are no longer available for biomass synthesis. A good gene knockout strategy should improve DHA yields substantially with a modest decrease in biomass growth rate.

**FIGURE 7:**
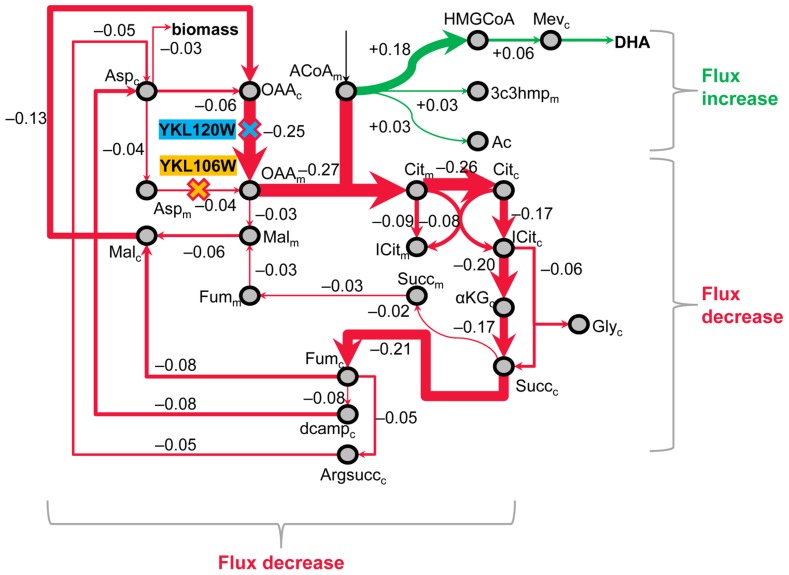
**MOMA analysis identifies double-gene knockouts in primary metabolism that funnel carbon toward DHA.** As per one MOMA prediction, engineering the Fy_DHA_ strain by knocking out two genes (**1:** *OAC1* or *YKL120W*, which codes for an oxaloacetate transporter and **2:** *AAT1* or *YKL106W*, which codes for an aspartate aminotransferase) should result in 4.2 mol DHA per 100 mol of glucose, which amounts to a ~70-fold increase as compared to our current yield and translates to ~18% of the theoretical yield of DHA from glucose. This metabolic map depicts the mechanism by which these two deletions accomplish the flux redirection. Red arrows indicate decreased flux and green arrows indicate increase in flux relative to our (control) DHA-producing strain; arrow thicknesses and numbers alongside the arrows represent flux differences between the baseline strain Fy_DHA_’ and the double-gene knockout strain are in arbitrary units. On the basis of the ^13^C isotopomer results, FBA predicted that our control strain loses a significant amount of mitochondrial acetyl-CoA (ACoA_m_) to CO_2_ through a cyclic, cytosolic pathway that contains most steps of the TCA cycle. Knocking out *YKL120W* and *YKL106W* breaks this cycle while not significantly affecting biomass production, thus allowing ACoA_m_ to be diverted toward downstream pathways including DHA biosynthesis.

The best double-gene knockout strategy predicted by MOMA includes the genes *YKL120W* and *YKL106W*, and improves DHA yield to 4.2 mol % (70-fold over the baseline), while reducing biomass yield by only 2.2-fold compared to Fy_DHA_’. **Figure 7** presents an interpretation of this strategy. First, FBA predicted that Fy_DHA_’ loses a significant amount of mitochondrial acetyl-CoA to CO_2_ through a cyclic, cytosolic pathway that contains most steps of the TCA cycle. Both *YKL120W* and *YKL106W* code for enzymes or transporter proteins in facilitating this cycle. *YKL120W* or *OAC1* codes for an oxaloacetate transporter catalyzing the transport oxaloacetate[c] + H^+^[c] ≫ oxaloacetate[m] + H^+^[m], where [c] and [m] denote the cytosol and mitochondrion, respectively. *YKL106W* or *AAT1* codes for an aspartate aminotransferase catalyzing the reaction α-ketoglutarate[m] + L-aspartate[m] ≫ L-glutamate[m] + oxaloacetate[m]. Thus, knocking out *YKL120W* and *YKL106W* breaks this cycle while not affecting biomass production, allowing mitochondrial acetyl-CoA to be diverted toward downstream pathways including DHA biosynthesis. Of these two genes, *YKL120W* has a greater effect on reducing flux toward mitochondrial oxaloacetate, and also shows up as part of the next best double-gene knockout pair for improving DHA yield (Data Sheet 3 in Supplementary Material).

A limitation of using FBA-type methodologies to predict genetic engineering strategies is their reliance of the assumption of optimized metabolism to predict flux distributions. However, engineered strains may perform sub-optimally. Thus, while FBA is valuable in identifying non-trivial gene targets, it is ambitious to expect the real engineered strain to exhibit yields close to the FBA-predicted yields. Additionally, a recent statistical analysis of previous studies on the production of chemicals by engineered *S. cerevisiae* found that genetic intervention improved product yield only by two- to fourfold in a majority of the 40 studies examined, and that chemicals requiring multiple enzymatic steps were associated with lower yields (Varman et al., 2011). Along the same lines, implementation of the best gene knockout strategy suggested by our study may result in a DHA yield improvement tangibly lower than the predicted 70-fold increase. Very likely, one round of genetic engineering in accordance with FBA predictions may necessitate further rounds of engineering to overcome further bottlenecks to product yield.

## CONCLUSION

In summary, we investigated various *in silico* and *in vivo* approaches – ExPa analysis, ^13^C MFA, FBA, and MOMA analysis – to identify metabolic engineering strategies for improving DHA yield in an engineered yeast strain. Our analyses revealed three major metabolic engineering strategies toward this objective. The first, most obvious strategy is reduction of flux from pyruvate to ethanol and directing this flux toward acetyl-CoA by knocking out fermentative pathways and overexpressing pyruvate dehydrogenase. However, in the absence of further genetic intervention, the available carbon is not likely to be redirected toward the desired pathway. A thorough investigation of the system using detailed ^13^C MFA, and FBA and MOMA analysis was required to decipher further non-intuitive strategies. A second strategy toward higher DHA yield is the improvement of NADPH availability by overexpressing *ME2* or a rate-limiting step of the oxidative PPP such as *G6PDH2*. Harnessing the oxidative PPP for NADPH supply suffers from the disadvantage that a sixth of the carbon entering this pathway is lost as CO_2_; hence, this lowers the yield of DHA significantly. A third strategy is pushing flux from mitochondrial acetyl-CoA toward DHA production. These two strategies are necessary for overcoming the NADPH and mitochondrial acetyl-CoA availability bottlenecks *en route* to DHA biosynthesis. We anticipate that comprehensive analysis of a similar type can be applied to any metabolic system to reveal intuitive and non-intuitive genetic engineering targets for optimizing the production of a target molecule. Such systemic analysis is an important step toward realizing the goal of rational metabolic engineering.

## CONTRIBUTIONS

Ashish Misra, Matthew F. Conway, Eddy C. Agbo, and Ganesh Sriram conceived this work; Ashish Misra, Matthew F. Conway, Joseph Johnnie, and Ganesh Sriram designed it. Ashish Misra, Matthew F. Conway, and Joseph Johnnie performed computational analyses; Matthew F. Conway and Ashish Misra performed ^13^C labeling experiments and ^13^C MFA. Tabish M. Qureshi performed RT-PCR and DHA assays. Ashish Misra and Ganesh Sriram interpreted the results, prepared data displays, wrote the manuscript and revised the manuscript.

## Conflict of Interest Statement

Eddy C. Agbo, Anne M. Derrick and Bao Lige are employed with Fyodor Biotechnologies Corp., which has financial interest in the commercialization of antimalarial therapeutics or their precursors. The other authors have no conflict of interest.

## Supplementary Material

The Supplementary Material for this article can be found online at http://www.frontiersin.org/Microbiotechnology,_Ecotoxicology_and_Bioremediation/10.3389/fmicb.2013.00200/abstract
